# Relapse in Tolosa-Hunt syndrome: pooled recurrence rates and associated factors from a meta-analysis of 456 cases

**DOI:** 10.1007/s10072-026-08985-7

**Published:** 2026-03-26

**Authors:** Bernardo Leite Pondé da Luz, Guilherme Diogo Silva

**Affiliations:** Division of Neurology, Hospital das Clínicas HCFMUSP, Faculdade de Medicina, Universidade de São Paulo, Avenida Dr. Enéas de Carvalho Aguiar, 255, Cerqueira César, São Paulo, SP Brazil

**Keywords:** Tolosa-Hunt syndrome, Recurrence, Risk factors, Systematic review, Relapse

## Abstract

**Objective:**

Tolosa-Hunt Syndrome (THS) is a rare, painful ophthalmoplegia caused by granulomatous inflammation within the cavernous sinus. Despite the effective response to corticosteroid therapy, recurrence rates reported in the literature vary significantly, and clear associated factors of recurrence remain elusive. This systematic review and meta-analysis aim to establish the frequency of relapse and identify f actors associated with recurrence.

**Evidence acquisition:**

Following PRISMA guidelines, we performed a comprehensive database search of MEDLINE/PubMed, EMBASE, and Scopus from inception to June 2024, focusing on observational studies or case series reporting THS recurrence. Meta-analysis employed a random-effects model to estimate pooled recurrence rates. Meta-regression analyses evaluated clinical and demographic associated factors of recurrence, with publication bias assessed via funnel plots and Egger’s test.

**Results:**

Seventeen studies encompassing 456 patients met the inclusion criteria. The pooled recurrence rate was 23% (95% CI, 17–32%), with substantial heterogeneity (I^2^ = 59.9%). The frequency of cranial nerve involvement specifically nerves II, IV, and VI, correlated with higher recurrence risk (*P* < 0.05). Age, sex, duration of follow-up, and MRI abnormalities did not show significant associations. Limited data were available concerning the use of steroid-sparing immunosuppressants, inflammatory biomarkers, and characteristics of MRI.

**Conclusion:**

Approximately one-quarter of patients with THS experience recurrence. Involvement of cranial nerves II, IV, and VI was associated with relapses. Further prospective investigations with standardized recurrence definitions and structured therapeutic comparisons are needed to clarify prognostic factors and optimize long-term management.

**Supplementary Information:**

The online version contains supplementary material available at 10.1007/s10072-026-08985-7.

## Introduction

Tolosa-Hunt Syndrome (THS) is a rare neurological disorder characterized by orbital pain associated with paresis of one or more ocular motor cranial nerves due to idiopathic granulomatous inflammation of the cavernous sinus, superior orbital fissure, or orbital apex [1]. First described by Tolosa in 1954 and later by Hunt et al. in 1961, THS is classically responsive to corticosteroids, often with rapid pain relief within 72 h, though ophthalmoplegia typically resolves more gradually [2, 3].

Although THS is usually considered a benign, self-limited condition, its course can be heterogeneous [4]. THS is notably associated with highly variable and unpredictable recurrence rates, ranging widely in the literature from approximately 9% to greater than 70% [5–8]. The timing and frequency of recurrence vary substantially, sometimes occurring years after the initial episode [7]. Recurrent cases often respond to corticosteroids but may require prolonged or repeated treatment, raising concerns about cumulative steroid exposure and quality-of-life impairment [9]. Despite reports in the literature, predictors of recurrence remain unclear. Studies have examined associations with age, sex, specific cranial nerve involvement, and imaging findings, but results are inconsistent [7, 9, 10]. Furthermore, data on the use of steroid-sparing immunosuppressants are limited and conflicting [7, 9]. Notably lacking in current literature are robust systematic analyses that synthesize existing data to clearly identify consistent clinical, demographic, or radiological predictors of relapse.

Given the heterogeneity in recurrence rates and the absence of consensus on risk factors, we conducted a systematic review and meta-analysis to (1) estimate the pooled recurrence rate of THS and (2) evaluate clinical, demographic, and radiological features potentially associated with relapse. Clarifying these aspects may help clinicians better counsel patients, tailor follow-up strategies, and better inform patient counseling after the initial episode.

## Methods

This systematic review was conducted in accordance with the Preferred Reporting Items for Systematic Reviews and Meta-Analyses (PRISMA) guidelines [11]. The protocol was registered on the international prospective register of systematic reviews PROSPERO (CRD42024628603).

A comprehensive search was performed across three electronic databases: MEDLINE/PubMed, EMBASE, and Scopus. The search used synonyms of the key terms “Tolosa-Hunt syndrome,” “recurrence,” and “risk factors”. We screened texts from inception to June 15th, 2024.

We included: 1) observational studies or case series; 2) with at least five patients diagnosed with THS based on the International Headache Society diagnostic criteria [12]; and 3) studies reporting relapses of THS. We excluded reviews, editorials, comments, and animal studies.

Two authors independently screened for eligibility in titles and abstracts, followed by full-text assessments. Discrepancies were resolved through consensus. We screened the reference lists of eligible articles to identify more manuscripts.

When multiple publications from the same cohort were identified, the version most aligned with the primary objective of estimating overall recurrence frequency and baseline clinical characteristics was included in the quantitative synthesis. Subsequent publications from the same cohort focusing primarily on therapeutic comparisons were excluded from the meta-analysis to avoid duplication and to preserve homogeneity of outcome definitions.

Two authors independently extracted data using a standardized form. We collected authors, publication year, number of patients, mean age, age standard deviation, gender distribution, affected cranial nerves (III [oculomotor], IV [trochlear], VI [abducens], V [trigeminal], II [optic]); abnormal neuroimaging findings (e.g., MRI-detected cavernous sinus abnormalities), steroid use, use of other immunosuppressants, number of recurrent cases, and mean follow-up duration in years. When reporting the minimal follow-up duration instead of the mean follow-up duration, we considered the minimal follow-up as the follow-up duration.

Recurrence definitions were extracted from each included study as originally reported. Across the 17 cohorts, operational criteria were not standardized. Nine studies reported recurrent episodes without providing a detailed operational definition beyond clinical relapse. Four studies defined recurrence clinically as the reappearance of periorbital pain and/or cranial nerve dysfunction after partial or complete remission. Three studies explicitly described relapse occurring after corticosteroid tapering or discontinuation. Only one study systematically documented recurrence with radiological correlation; however, no study defined relapse based solely on imaging progression in the absence of clinical deterioration. Case series were interpreted as having a high risk of bias due to their retrospective design and absence of control groups. To address duplicate reporting, studies with overlapping populations or suspected data replication were excluded.

We presented a pooled estimate of the proportion of patients who experienced relapses of THS using a random-effects meta-proportion model. Heterogeneity was estimated using the I^2^ statistic, and we explored sources of heterogeneity through meta-regression analyses of each extracted variable. In addition, subgroup analyses were conducted to further investigate potential contributors to between-study heterogeneity. Studies were stratified according to follow-up duration (≥ 2 years vs < 2 years), sample size (≥ 20 vs < 20 patients), and clarity of relapse definition (explicit clinical definition vs not specified). Pooled estimates and heterogeneity measures were calculated within each subgroup.

Reporting bias was assessed by visually examining the symmetry of a funnel plot, complemented by Egger's test for funnel plot asymmetry.

## Results

### Study selection and demographics

**Fig. 1:**
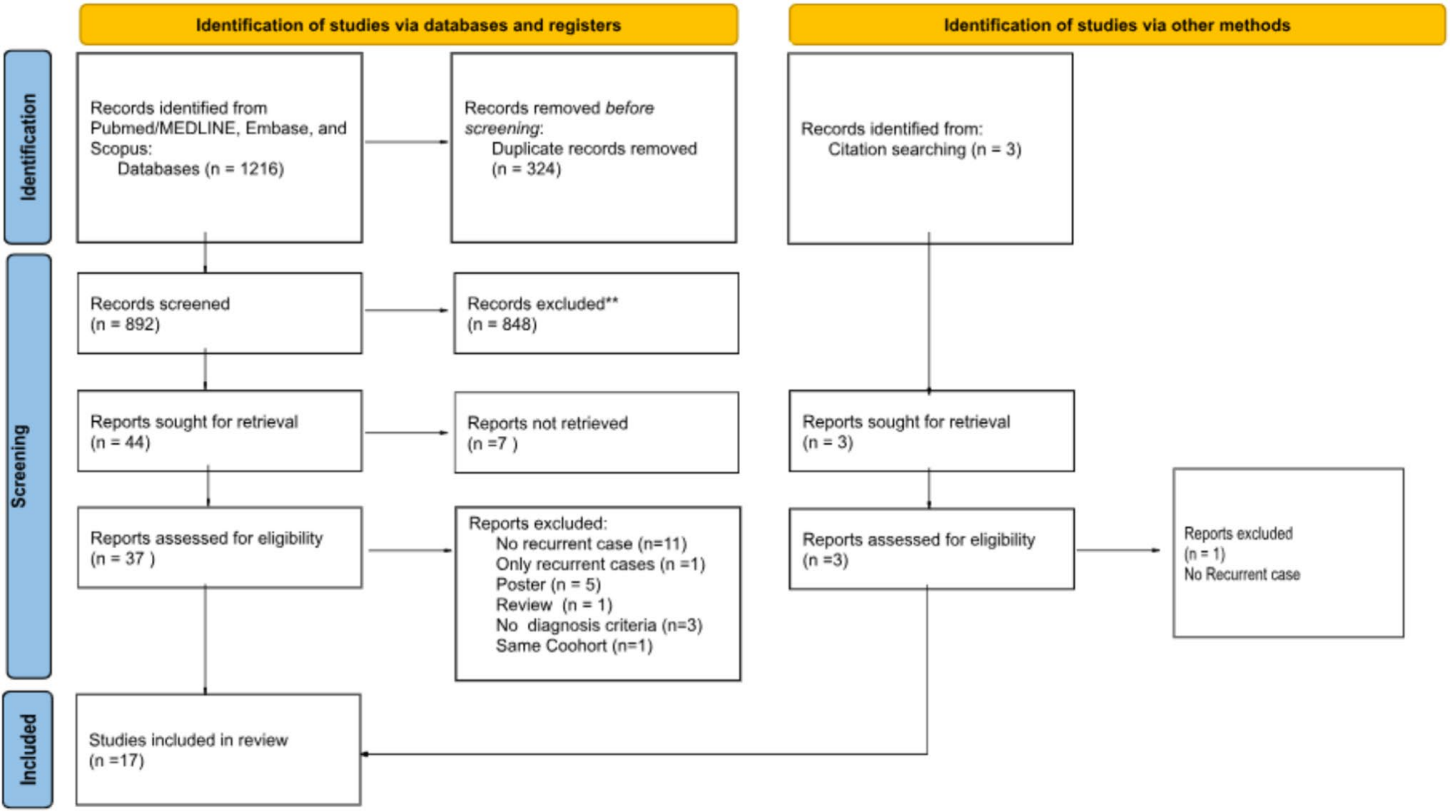
Flow diagram

We included 17 studies with a total of 456 patients (Fig. 1). Study characteristics are presented in Table 1.

**Table 1:** Characteristics of studies reporting relapsing Tolosa-Hunt syndrome

Study	*n*	Country	Relapse (%)	Follow up	Mean age ± SD	Male (*n*)	III	IV	VI	V	II	Altered MRI	Steroid
Ertilav [13]	11	Turkey	18	2	-	-	-	-	-	-	-	8	11
Rodge [14]	73	India	11	1	-	-	70	69	65	65	35	72	73
Kim [9]	91	South Korea	27	2,1	40 ± 14,9	48	54	15	43	14	5	91	25
Ata [8]	31	Qatar	10	1	40 ± 12	22	22	8	17	4	-	20	3
Arthur [7]	44	India	41	3,25	49,5 ± 13	23	44	7	7	22	7	40	44
Podgorac [5]	8	Serbia	62	2,05	53,6 ± 86,7	2	7	4	6	4	-	5	8
Akpinar [6]	7	Turkey	71	2,3	45,7 ± 18,2	4	5	3	3	1	-	7	4
Peréz [15]	13	USA*	23	-	10,5 ± 3,53	-	11	2	7	1	-	9	12
Bhatkar [16]	17	India	12	0,5	-	-	-	-	-	-	-	-	17
Zhang [17]	46	China	37	0,83	44,4 ± 15,6	28	36	14	19	13	5	24	17
Hao [18]	22	China	23	1	-	9	20	20	15	6	2	20	5
Hung [10]	49	Taiwan	8	-	52,1 ± 16,4	20	42	33	29	-	21	23	4
Lee [19]	6	South Korea	33	2,41	44,8 ± 10,3	3	5	1	3	1	-	6	2
Jain [20]	7	Oman	14	0,3	38 ± 18,6	5	4	2	3	-	-	7	1
Turkoglu [21]	10	Turkey	30	3,85	44 ± 18,5	6	6	1	6	4	2	9	3
Fernandéz [22]	16	Spain	19	4,49	56,7 ± 18	6	14	8	10	8	-	4	14
Carrilho [23]	5	Brazil	20	-	51 ± 15,15	0	5	2	1	4	-	-	1

*n* number of patients with THS, *Relapse* number of patients that had at least 1 recurrence, *Follow up* mean follow up in years, *Age(mean)* mean age of patients with HS, *Age(sd)* age standard deviation of patients with THS, *Male* number of male patients, *III* number of patients with III nerve dysfunction, *IV* number of patients with IV nerve dysfunction, *VI* number of patients with VI nerve dysfunction, *V* number of patients with V nerve dysfunction, *II* number of patients with II nerve dysfunction, *Altered MRI* number of patients with altered MRI compatible with THS, *Other treatment* number of patients that used other immunosuppressant medication other than corticosteroids; * this paper is from USA but it's population is from multiple origins

The countries of origin for the studies and their respective populations are detailed in Table 1. Out of a total of 456 patients, 135 were from India, 119 were of Chinese descent (from China and Taiwan), 97 were from South Korea, 39 were from the Middle East (Qatar and Oman), and 28 were from Turkey. Figure 1 represents the country of origin of the included manuscript. The remaining patients were from various countries, including Spain (17), Serbia (8), Brazil (5), the USA (2), Belgium (1), Poland (1), Italy (1), Japan (1), Hungary (1), and Uruguay (1).

### Relapse rates and associated factors

**Fig. 2:**
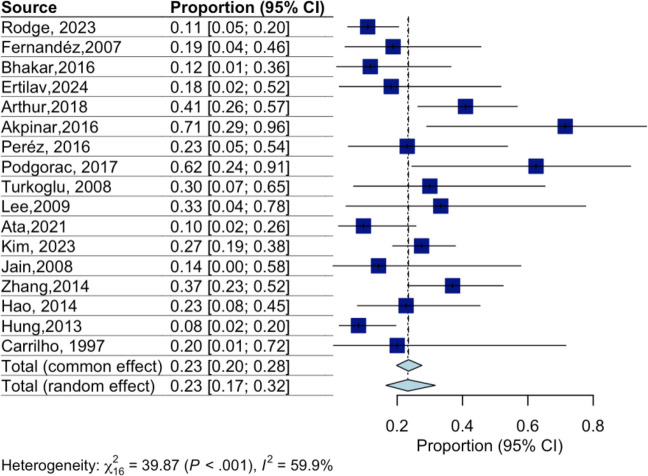
Forest plot of the proportion of patients who experienced relapse of Tolosa-Hunt syndrome. Horizontal lines represent 95% confidence intervals (CI) for the individual study proportions. The squares indicate point estimates, with their size proportional to the weight of the study in the meta-analysis. The diamond represents the pooled proportion and its 95% CI using a random-effects model. Heterogeneity: χ^2^ = 39.87 (*P* < 0.001), I^2^ = 59.9%

The pooled estimated relapse rate was 23% (95% CI 17–32%, I^2^ = 59.9%) (Fig. 2). Recurrence proportions varied substantially across individual studies, ranging from approximately 10% to over 60%, with the highest rates reported in small single-center cohorts.

In meta-regression analysis, involvement of cranial nerves IV, VI, and II was associated with recurrence, whereas age, gender, and abnormal magnetic resonance imaging were not significant moderators (Table 2).

**Table 2:** Meta-regression for associated factors of heterogeneity

Characteristic	Test of moderators (*P*-value)	Estimate
Age	0.69	0.01
Sex (% of males)	0.65	- 0.01
III nerve involvement	0.19	- 0.01
IV nerve involvement	0,01*	- *0.02*
V nerve involvement	0.09	- 0.02
VI nerve involvement	0.04*	- *0.02*
II nerve involvement	< 0.01*	- *0.05*
Altered magnetic resonance imaging	0.56	- 0.00
Follow-up duration	0.15	0.25

Characteristics evaluated include patient demographics (age, sex) and clinical factors (cranial nerve involvement, altered magnetic resonance imaging findings, and follow-up duration). The test of moderator’s column shows *P*-values, with statistically significant values indicated by (*). The estimate column represents the magnitude and direction of the effect for each predictor. Significant predictors include IV nerve involvement (*P* = 0.01), VI nerve involvement (*P* = 0.04), and II nerve involvement (*P* < 0.01)

Subgroup analyses demonstrated that studies with follow-up ≥ 2 years showed higher recurrence estimates and reduced heterogeneity (I^2^ = 42.5%). In contrast, stratification by sample size (≥ 20 patients) and by clarity of relapse definition did not substantially reduce between-study variability (Online resources 1–3).

### Publication bias analysis

**Fig. 3:**
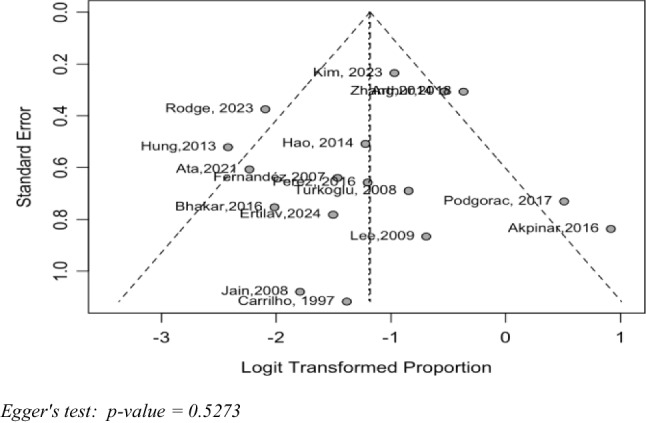
Funnel plot. Egger's test: *p*-value = 0.5273

The funnel plot analysis (Fig. 3) and Egger's test (*p* = 0.5273) did not indicate significant evidence of publication bias.

## Discussion

In this systematic review of 456 patients with Tolosa–Hunt syndrome (THS), we found a pooled relapse frequency of approximately 23%. This finding underscores that, although THS generally responds well to corticosteroids [1], nearly one in four patients will experience a recurrence. Notably, we identified a significant association between II, IV, and VI cranial nerve involvement and the likelihood of relapse. In contrast, no significant associations with recurrence were observed for patient age, sex, overall follow-up duration, or the presence of MRI abnormalities at baseline.

The variability in reported recurrence rates across studies highlights the methodological and clinical heterogeneity of the available literature. Recurrence proportions ranged from < 10% to > 60% across cohorts. Akpinar et al. reported a 71% recurrence rate [6]; three of the five relapsing patients were subsequently reclassified as misdiagnosed cases, and the remaining two had not received maintenance therapy. Podgorac et al. observed a 62% recurrence rate, predominantly during corticosteroid tapering, while Arthur et al. reported a recurrence rate of 48.6% in a longitudinal cohort with extended follow-up [5, 7]. In contrast, Ata et al. described a recurrence rate of 9% in a limited cohort, and a study from Eastern India reported an 11% rate [8, 14]. The studies reporting the highest recurrence proportions differed from others in several methodological aspects. Two were small case series, increasing statistical variability and susceptibility to extreme proportions, and all three were single-center cohorts with detailed longitudinal follow-up and specific attention to disease evolution. However, prolonged follow-up alone does not fully explain variability, as other cohorts with extended observation periods reported more moderate recurrence rates. Recurrence detection, therefore, appears to be influenced by a combination of factors, including sample size, study focus, referral patterns, therapeutic strategies, and operational definitions of relapse. Heterogeneity in the criteria used to define recurrence across studies likely further contributed to differences in reported recurrence proportions.

Despite the common perception of THS as a benign condition, our findings demonstrate that a significant proportion of patients experience disease recurrence, which may lead to long-lasting neurological disability. Nearly all patients in the included studies were treated with corticosteroids, which typically yield rapid pain relief within 72 h, as per ICHD-3 criteria [12]. However, ophthalmoplegia often persists, with complete resolution rates ranging from 61 to 95% [10, 17, 18, 24]. Inzitari et al. [24] reported that corticosteroids did not significantly shorten the duration of paresis. Hung et al. [10] observed complete pain relief but only an 80% resolution of diplopia. Zhang et al. [17] reported partial or complete resolution of paresis in most patients by eight months, but only a small proportion experienced full recovery within a week. Additionally, therapeutic response during recurrence episodes was often diminished, requiring higher corticosteroid doses [17]. This not only prolongs patient morbidity but also contributes to increased socioeconomic costs and a heightened cumulative exposure to corticosteroids, with their associated risks.

In our meta-regression analysis, involvement of cranial nerves II, IV, and VI was associated with higher recurrence rates. This finding should be interpreted cautiously. The observed associations may reflect greater disease extent or severity rather than intrinsic characteristics of specific nerves. However, given the aggregate nature of the data and the absence of individual imaging-level correlation, no mechanistic inference can be drawn from this analysis. Furthermore, as the analysis was conducted at the study level rather than using individual patient data, these associations may be subject to ecological bias.

Our study’s average follow-up was 1.83 years, during which most recurrences occurred within the first year, suggesting incomplete resolution of the initial inflammatory process. Nonetheless, recurrences up to 13 years post-diagnosis have been reported [25]. Short mean follow-up durations (0.3 to 4.49 years) in the included studies may have hindered our ability to detect long-term recurrence patterns [20, 22]. Although follow-up time is suspected to influence recurrence risk, it was not identified as a statistically significant moderator in the meta-regression analysis. However, in predefined subgroup analyses, studies with follow-up ≥ 2 years demonstrated higher pooled recurrence estimates and reduced heterogeneity (I^2^ = 42.5%), suggesting that shorter observation periods may underestimate the true recurrence frequency. In contrast, stratification by sample size and clarity of relapse definition did not meaningfully reduce between-study variability (see Online Resources 1–3). These findings indicate that differences in longitudinal assessment may partially account for heterogeneity across cohorts and highlight the need for standardized follow-up strategies in future studies.

Some authors have proposed subtypes of THS: "benign" THS with normal MRI and "inflammatory" THS with visible granulomas, the latter aligning with ICHD-3 criteria [10]. Although inflammatory THS has been linked to higher recurrence [10], our review did not confirm this due to insufficient imaging detail data across studies. We consider that it is important that future studies compare recurrence rates based on specific alterations on MRI, such as lesion size. Future research should also explore the prognostic value of inflammatory biomarkers such as C-reactive protein (CRP), erythrocyte sedimentation rate (ESR), and cerebrospinal fluid profiles. While Arthur et al. [7] found elevated CRP/ESR in 34% of patients (with a 41% recurrence rate), Hung et al. [10] reported grossly normal markers and only an 8% recurrence rate. These markers warrant further investigation as potential prognostic indicators in future prospective studies.

Corticosteroids remain the mainstay of THS treatment, although optimal dosing and tapering strategies are not standardized. While some studies reported shorter duration of ophthalmoplegia with high-dose intravenous therapy, no consistent reduction in recurrence rates has been demonstrated [4, 7, 9]. Due to limited reporting and heterogeneity in treatment protocols, corticosteroid dosing and use of additional immunosuppressive agents were not included in the meta-regression analysis. Two studies explored the use of additional immunosuppressive agents. In a subsequent therapeutic analysis derived from the same institutional cohort included in this review, Arthur et al. [26] reported that patients receiving adjunctive steroid-sparing immunosuppressants had a lower relapse rate (20% vs 53.8%, *P* < 0.034). As this later publication represents an expanded therapeutic sub analysis of the previously reported cohort and was not included in the quantitative synthesis to avoid duplication, these data were not incorporated into the pooled analysis. In contrast, Kim et al. [9] in a cohort of 91 patients, found no statistically significant difference in recurrence rates between patients treated with steroid-sparing agents and those managed with corticosteroids alone.

This review has several limitations. Therapeutic data were inconsistently reported across included cohorts, and only two studies described the use of steroid-sparing immunosuppressive agents, precluding quantitative evaluation of treatment-related effects on recurrence. Attempts to obtain individual patient data were unsuccessful, restricting more granular analyses. In subgroup analyses, studies with follow-up duration of at least two years demonstrated lower heterogeneity (I^2^ = 42.5%) and higher pooled recurrence estimates, suggesting that shorter observation periods may have led to underestimation of recurrence frequency in some cohorts. Finally, a substantial proportion of included studies originated from Asian populations, which may limit the generalizability of these findings to other regions and healthcare settings.

Our study’s strengths include a substantial sample size and a comprehensive analysis of recurrence risk factors. Moreover, assessment of publication bias via funnel plot analysis suggests a low likelihood of selective reporting.

## Conclusion

This systematic review provides the largest pooled estimate of recurrence in Tolosa–Hunt syndrome to date, consolidating data from 456 patients across 17 studies. Although THS is traditionally regarded as a benign and self-limited condition, our findings indicate that approximately one quarter of patients experience at least one recurrence, with individual study estimates ranging from approximately 10% to over 60%. This substantial heterogeneity was not fully explained by the investigated study-level characteristics, although follow-up duration and cranial nerve involvement pattern may have partially contributed. Future prospective multicenter studies are needed to better characterize the sources of variability in recurrence rates, validate the prognostic role of cranial nerve involvement, and establish evidence-based long-term management strategies.

## Supplementary Information

Below is the link to the electronic supplementary material.

Supplementary file1 (DOCX 236 KB)

## References

[1] Tolosa E (1954) Periateritic lesions of the carotid siphon with the clinical features of a carotid infraclinodal aneurysm. J Neurol Neurosurg Psychiatry 17(4):300–302. 10.1136/jnnp.17.4.300 13212421 10.1136/jnnp.17.4.300 PMC503202

[2] Hunt WE, Meagher JN, LeFever HE, Zeman W (1961) Painful ophthalmoplegia: its relation to indolent inflammation of the cavernous sinus. Neurology 11(1):56–56. 10.1212/WNL.11.1.56 13716871 10.1212/wnl.11.1.56

[3] Smith JL, Taxdal DS (1966) Painful ophthalmoplegia. The Tolosa-Hunt syndrome. Am J Ophthalmol 61(6):1466–1472. https://pubmed.ncbi.nlm.nih.gov/5938314/. Accessed 17 July 2024 5938314

[4] Ahmed HS, Shivananda DB, Pulkurthi SR, Dias AF, Sahoo PP (2024) Clinical profile and outcomes in Tolosa-Hunt syndrome; a systematic review. J Clin Neurosci 129:110858. 10.1016/j.jocn.2024.110858 39366127 10.1016/j.jocn.2024.110858

[5] Podgorac A, Zidverc-Trajkovic J, Jovanovic Z et al (2017) Tolosa-Hunt syndrome: is it necessary to show granuloma? - The report of eight cases. Vojnosanit Pregl 74(3):287–293. 10.2298/vsp150703180p

[6] Akpinar ÇK, Özbenli T, Doğru H, Incesu L (2017) Tolosa-Hunt syndrome - cranial neuroimaging findings. Noro Psikiyatr Ars 54(3):251–254. 10.5152/npa.2016.13791 29033638 10.5152/npa.2016.13791 PMC5630104

[7] Arthur S et al (2018) Clinical profile and long-term outcome of Tolosa-Hunt syndrome. Ann Indian Acad Neurol 21(4):302–306. 10.4103/aian.AIAN_368_18 10.4103/aian.AIAN_368_18 PMC7061504 32189862

[8] Ata F, Yousaf Z, Arachchige SNM, Rose S, Alshurafa A, Muthanna B, Bilal AIB, El Beltagi AE, Zahid M (2021) The demographics of Tolosa-Hunt syndrome in Qatar. eNeurologicalSci 24:100359–100359. 10.1016/j.ensci.2021.100359 34355072 10.1016/j.ensci.2021.100359 PMC8325092

[9] Kim H, Oh SY (2021) The clinical features and outcomes of Tolosa-Hunt syndrome. BMC Ophthalmol 21(1):237. 10.1186/s12886-021-02007-0 34044807 10.1186/s12886-021-02007-0 PMC8161661

[10] Hung CH, Chang KH, Wu YM, Chen YL, Lyu RK, Chang HS, Wu YR, Chen CM, Huang CC, Chu CC, Liao MF, Wai YY, Hsu SP, Ro LS (2013) A comparison of benign and inflammatory manifestations of Tolosa-Hunt syndrome. Cephalalgia 33(10):842–852. 10.1177/0333102412475238 23475292 10.1177/0333102412475238

[11] Page MJ, McKenzie JE, Bossuyt PM, Boutron I, Hoffmann TC, Mulrow CD, Shamseer L, Tetzlaff JM, Akl EA, Brennan SE, Chou R, Glanville J, Grimshaw JM, Hróbjartsson A, Lalu MM, Li T, Loder EW, Mayo-Wilson E, McDonald S, McGuinness LA, Stewart LA, Thomas J, Tricco AC, Welch VA, Whiting P, Moher D (2021) The PRISMA 2020 statement: an updated guideline for reporting systematic reviews. BMJ. 10.1136/bmj.n71 33782057 10.1136/bmj.n71 PMC8005924

[12] International Headache Society (2018) Headache classification committee of the international headache society (IHS) the international classification of headache disorders, 3rd edition. Cephalalgia 38(1):1–211. 10.1177/0333102417738202 10.1177/0333102417738202 29368949

[13] Ertilav E, Akyol A (2024) Evaluation of patients with painful ophthalmoplegia for benign and secondary etiologies. Neuro-Ophthalmology 48(5):338–347. 10.1080/01658107.2024.2336270 39145318 10.1080/01658107.2024.2336270 PMC11321404

[14] Rodge VN, Mukherjee A, Biswas S, Majumdar S, Gangopadhyay G (2023) Clinical and radiological profile of cavernous sinus syndrome: a study from eastern part of India. Egypt J Neurol Psychiatry Neurosurg. 10.1186/s41983-023-00667-x

[15] Pérez CA, Evangelista M (2016) Evaluation and management of Tolosa–Hunt syndrome in children: a clinical update. Pediatr Neurol 62:18–26. 10.1016/j.pediatrneurol.2016.06.017 27473647 10.1016/j.pediatrneurol.2016.06.017

[16] Bhatkar S, Goyal MK, Takkar A, Mukherjee KK, Singh P, Singh R, Lal V (2017) Cavernous sinus syndrome: a prospective study of 73 cases at a tertiary care centre in Northern India. Clin Neurol Neurosurg 155:63–69. 10.1016/j.clineuro.2017.02.017 28260625 10.1016/j.clineuro.2017.02.017

[17] Zhang X, Zhang W, Liu R, Dong Z, Yu S (2014) Factors that influence Tolosa–Hunt syndrome and the short-term response to steroid pulse treatment. J Neurol Sci 341(1–2):13–16. 10.1016/j.jns.2014.03.031 24703581 10.1016/j.jns.2014.03.031

[18] Hao R, He Y, Zhang H, Zhang W, Li X, Ke Y (2015) The evaluation of ICHD-3 beta diagnostic criteria for Tolosa–Hunt syndrome: a study of 22 cases of Tolosa–Hunt syndrome. Neurol Sci 36(6):899–905. 10.1007/s10072-015-2124-2 25736249 10.1007/s10072-015-2124-2

[19] Lee HK, Lee SG (2009) Clinical observations on tolosa-hunt syndrome. J Korean Ophthalmol Soc 50(11):1717. 10.3341/jkos.2009.50.11.1717

[20] Jain R, Sawhney S, Koul RL, Chand P (2008) Tolosa-hunt syndrome: MRI appearances. J Med Imaging Radiat Oncol 52(5):447–451. 10.1111/j.1440-1673.2008.01988.x 19032389 10.1111/j.1440-1673.2008.01988.x

[21] Türkoğlu R, Balak N, Tireli H (2008) Surgery with cavernous sinus syndrome. Neurosurg Q 18(4):230–238. 10.1097/wnq.0b013e31818d0980

[22] Fernández S, Godino O, Martínez-Yélamos S et al (2007) Cavernous sinus syndrome: a series of 126 patients. Medicine (Baltimore) 86(5):278–281. 10.1097/MD.0b013e318156c67f 17873757 10.1097/MD.0b013e318156c67f

[23] Carrilho E, Yamamoto FI, Scaff M (1997) Síndrome de Tolosa-Hunt: dificuldades no diagnóstico e padrão de resposta à prednisona. Arq Neuropsiquiatr 55(1):101–105. 10.1590/s0004-282x1997000100016 9332568 10.1590/s0004-282x1997000100016

[24] Inzitari D, Sit D, Marconi GP, Barontini F (1981) The tolosa-hunt syndrome: further clinical and pathogenetic considerations based on the study of eight cases. J Neurol 224(3):221–228. 10.1007/bf00313284 6162018 10.1007/BF00313284

[25] Giménez-Roldán S, Guillem A, Muñoz L (2006) Long-term risk of relapses in Tolosa-Hunt syndrome. Neurologia (Barcelona, Spain) 21(7):382–385. https://pubmed.ncbi.nlm.nih.gov/16977560/. Accessed 17 July 2024 16977560

[26] Arthur S et al (2020) Tolosa-hunt syndrome: long-term outcome and role of steroid-sparing agents. Ann Indian Acad Neurol 23(2):198–203. 10.4103/aian.aian_368_18 10.4103/aian.AIAN_368_18 PMC7061504 32189862

