# Supplementary material for: Relapse in Tolosa-Hunt syndrome: pooled recurrence rates and associated factors from a meta-analysis of 456 cases
Source: Neurol Sci. 2026 Mar 26;47(4):378. doi: 10.1007/s10072-026-08985-7 (PMC13021801; doi:10.1007/s10072-026-08985-7)
Supplement: Supplementary file 1 — Supplementary file1 (DOCX 236 KB) [file 10072_2026_8985_MOESM1_ESM.docx]

**Online Resource 1.** Subgroup analysis according to follow-up duration (≥2 years vs <2 years)

**
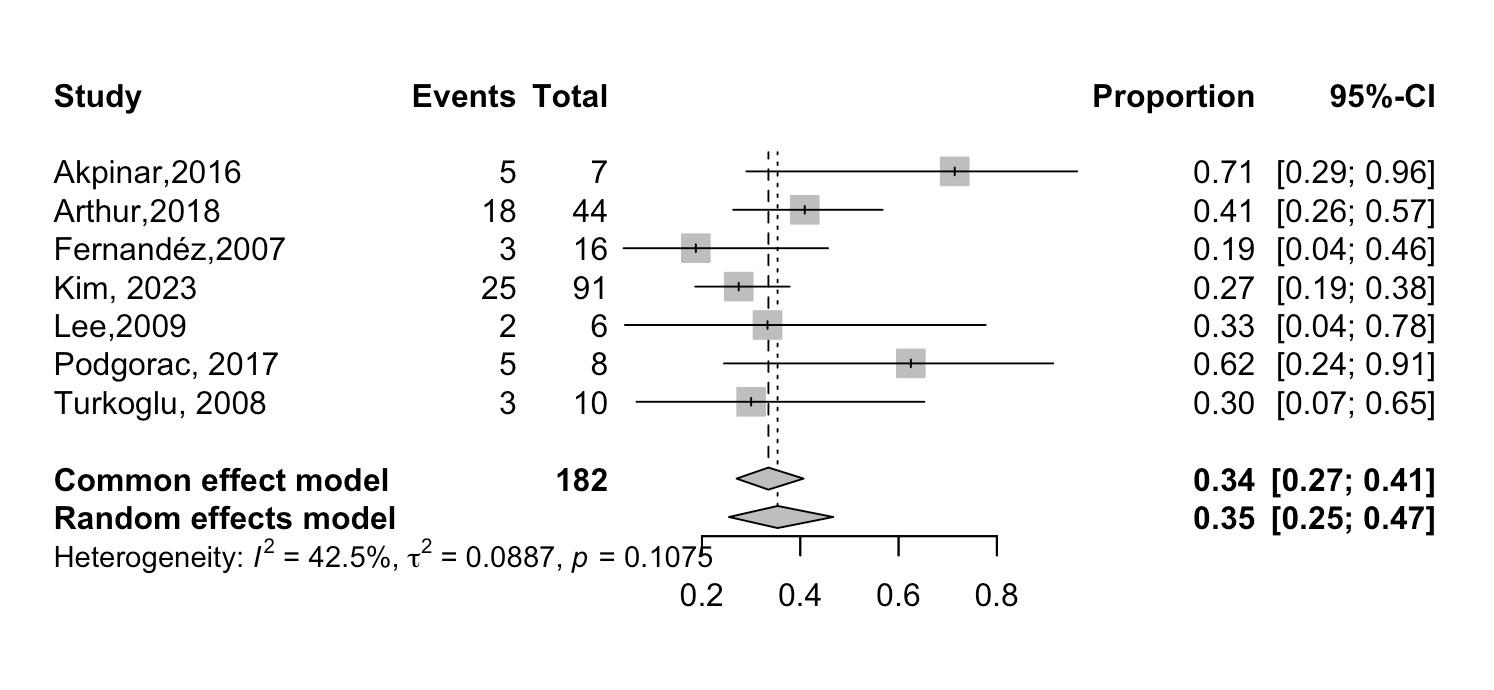
***Horizontal lines represent 95% confidence intervals (CI) for the individual study proportions. The squares indicate point estimates, with their size proportional to the weight of the study in the meta-analysis. The diamond represents the pooled proportion and its 95% CI using a random-effects model.*  *Heterogeneity: χ² = 0.0887 (P > 0.001), I² = 42.5%*

**Online Resource 2.** Subgroup analysis according to sample size (≥20 vs <20 patients)


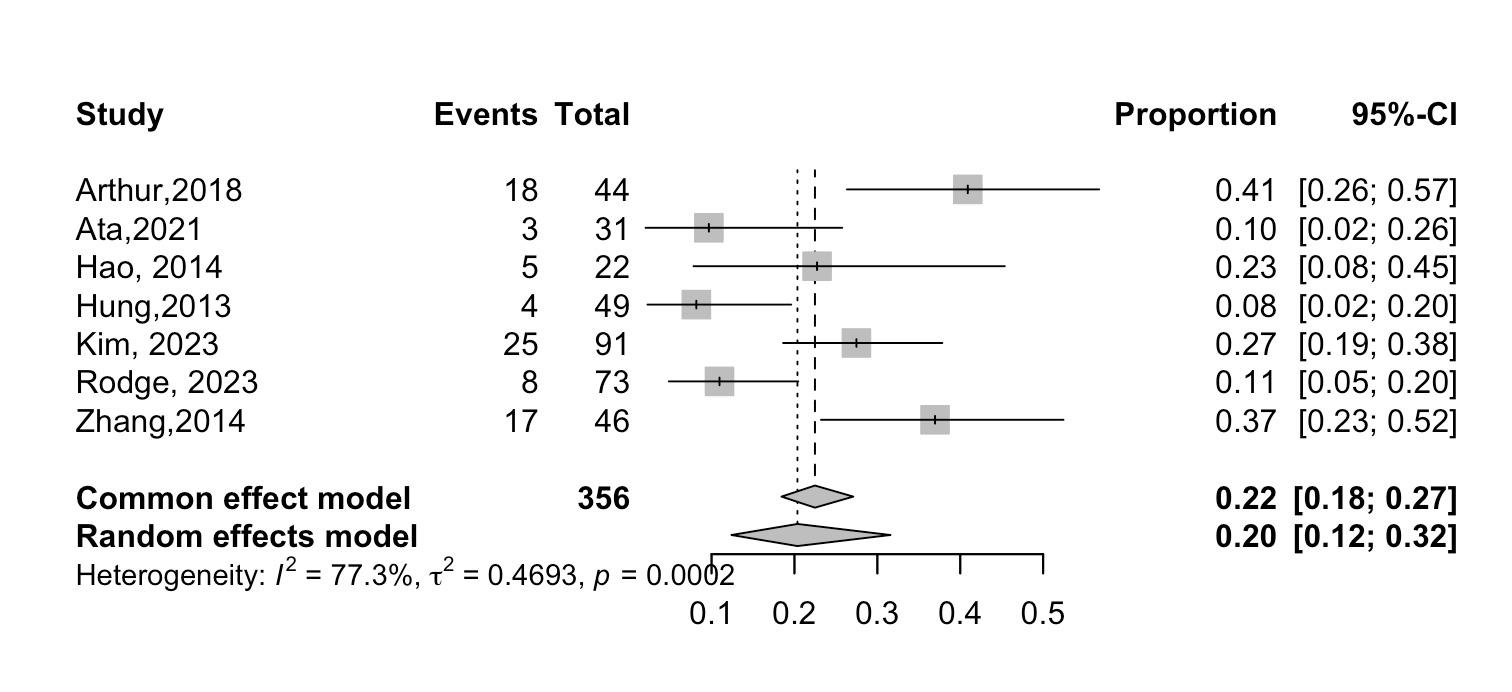
*Horizontal lines represent 95% confidence intervals (CI) for the individual study proportions. The squares indicate point estimates, with their size proportional to the weight of the study in the meta-analysis. The diamond represents the pooled proportion and its 95% CI using a random-effects model.*  *Heterogeneity: χ² = 0.4693 (P < 0.001), I² = 77.3%*

**Online Resource 3.** Subgroup analysis according to clarity of relapse definition


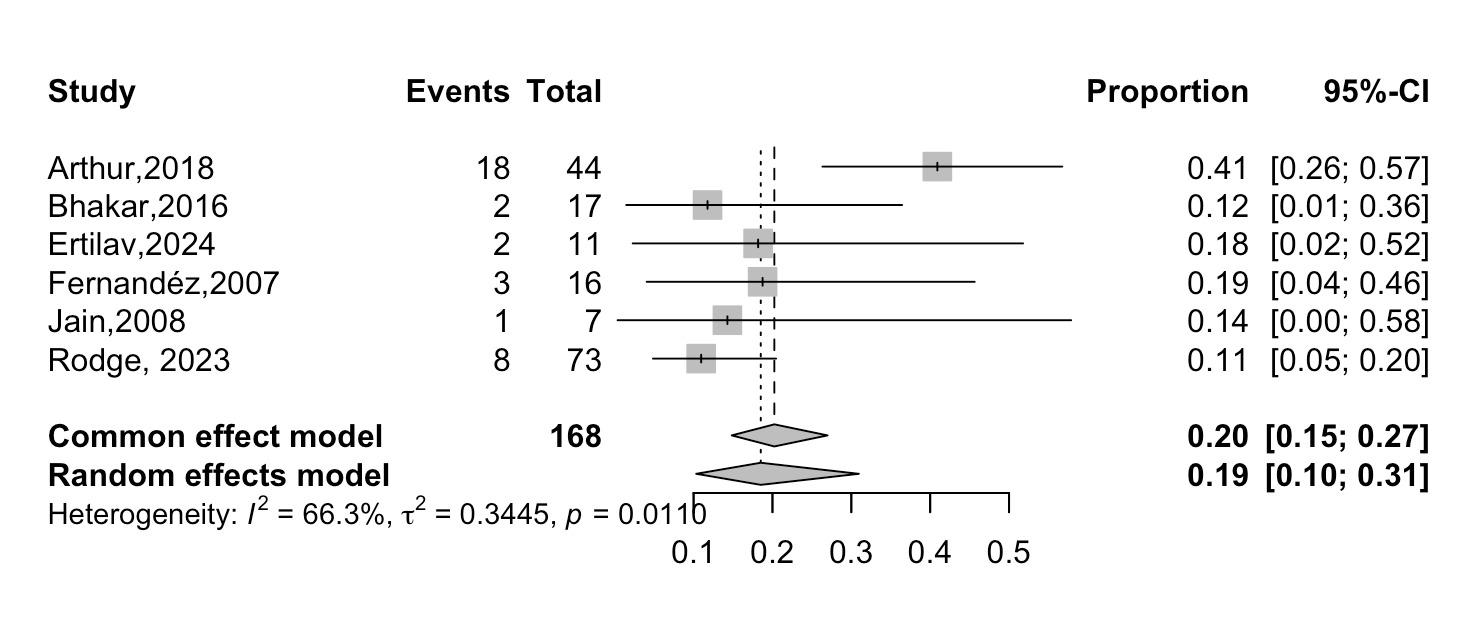


*Horizontal lines represent 95% confidence intervals (CI) for the individual study proportions. The squares indicate point estimates, with their size proportional to the weight of the study in the meta-analysis. The diamond represents the pooled proportion and its 95% CI using a random-effects model.*  *Heterogeneity: χ² = 0.3445 (P > 0.001), I² = 66.3%*
